# Application of Censored Quantile Regression to Determine Overall Survival Related Factors in Breast Cancer

**Published:** 2016-01-18

**Authors:** Javad Faradmal, Ghodratollah Roshanaei, Maryam Mafi, Abdolazim Sadighi-Pashaki, Manoochehr Karami

**Affiliations:** ^a^ Modeling of Noncommunicable Diseases Research Center, Department of Biostatistics, School of Public Health, Hamadan University of Medical Sciences, Hamadan, Iran; ^b^ Department of Biostatistics, School of Public Health, Hamadan University of Medical Sciences, Hamadan, Iran; ^c^ Radiology-Oncology Center, MRI Center of Hamadan Darol-Aitam, Hamadan, Iran; ^d^ Social Determinants of Health Research Center and Department of Epidemiology, School of Public Health, Hamadan University of Medical Sciences, Hamadan, Iran

**Keywords:** Breast Cancer, Survival Analysis, Quantiles

## Abstract

**Background:** The occurrence and the mortality related to breast cancer (BC) in Iranian female population has increased over time. Although there are many studies on BC and related risk factors, however, the epidemiological aspects of this melanoma in Iranian females are uncertain. Therefore, the aim of this study was to determine the relationship between demographical and clinical factors on the shape of overall survival (OS) distribution in patients with BC.

**Methods:** This historical cohort study was carried out using data from 522 participants with BC. Data were gathered from medical records of these patients admitted to Mahdieh Oncology Center of Hamadan Province, western Iran, from January 2000 to August 2011. Kaplan-Meier estimator was used to estimate the survival rates and, censored quantile regression (CQR) to provide in-depth insight in the multivariable association between prognosis factors and survival rates.

**Results:** Patients' follow-up ranged from around 3 to 197 months. One-, three-, and five-year survival rates were 90%, 73% and 62.5%, respectively. Results of CQR model showed that change in the age at diagnosis, number of involved lymph nodes and tumor size could significantly change the median and some other quantiles of OS.

**Conclusions:** This study, confirm the importance of early detection of BC and usefulness of CQR because of possible changes in distribution family of survival time.

## Introduction

In the recent decades, the disease related challenges in Iran have been changed from communicable diseases to noncommunicable diseases and car crash injuries^1^. In 2003, from 215.7 DALYs due to disease and injuries in Iran, 47% related to females^2^. Breast cancer (BC) with 24/100,000 cases is the most frequent cancer in Iranian women^3^ and comprises 24.4% of all malignants^3^. The occurrence of BC in Iranian female population increased from 12.6% in 1965 to 25.3% in 1998^4^ and its related mortality has increased over time^5^.

In the study related to treatment of malignancies, the overall survival (OS), quality of life and disease free survival (DFS) are the most interesting outcomes^6-7^. Different factors affect DFS or OS in patients with BC. Many studies have explored the relation between DFS or OS with demographical, tumor-related, genetics and treatment strategies of BC patients^8-11^. Accordingly, different models have been developed. Royston proposed the parametric lognormal model as a risk prediction model in cancer studies^12^. Farhadian et al., developed and applied a supervised wavelet method for predicting survival status of patients with BC^8^. Faradmal et al., applied and compared the results of artificial neural network with log-logistic model to predict recurrent of tumor in BC patients^13^. The interval coded score index for censored data modified to overcome the tradeoff between advanced modeling techniques and their interpretability was illustrated on prognosis of BC patients^14^.

All the above-mentioned models evaluated the effects of risk factors on the mean or median of the (function of) outcome. Besides, in the parametric survival models, it is assumed that the determined type of the outcome distribution do not change over time. To overcome the above-mentioned shortcomings, censored quantile regression (CQR) was developed^15-20^ as a valuable alternative to the parametric and semi-parametric models such as Cox proportional hazard model^18,21^. CQR has easy interpretations because of direct modeling of conditional quantiles^16,18,20-21^,and bring in considerable flexibility in assessing the relationship between risk factors and outcome^20^. Specially, the distribution-free property of CQR allows the change in the shape of distribution of outcome in subgroups.

Although there are many published studies on BC and related risk factors in Iranian women population,^4, 8^ but the epidemiological aspects of this melanoma in the above-mentioned population are uncertain^4^. Moreover, there are limited studies that explorer in depth the change in distribution of OS, related to its risk factors^15-21^. Therefore, the aim of this study was to introduce the CQR in survival analysis and to determine the relationship between demographical and clinical factors on the shape of OS distribution in Iranian BC population.

## Methods

### Data source and patients criteria

In this historical cohort study, data were gathered from medical records of BC patients admitted to Mahdieh Oncology Center of Hamadan Province, Iran, from January 2000 to August 2011. BC patients with the following criteria included: being a female, underwent breast conservative or (modified) radical mastectomy surgery, receiving chemotherapy and/or radiotherapy treatment after surgery. Finally, 522 patients (age ranged from 23 to 80 yr with mean ±SD age of 47.04 ±10.70) were enrolled.

### Survival time and prognosis factors

The primary aim was to determine the survival-associated predictors in BC. The (complete) survival time was defined as the duration (days/months) from surgery to death due to BC. It is possible that one or more patients experience death due to cause(s) other than BC or be alive at the end of the study. For these patients, the time from surgery to these end-points were considered as censored survival time. The date of surgery and end-point time for each patient was extracted from medical records or phone call by the investigators.

In addition to treatment strategies, patients and tumor characteristics were included as the predictors of time to death from BC. Demographics, tumor and treatment-related characteristics were age at diagnosis, family history, marriage status, tumor size, tumor histology, Her-2 and estrogen and progesterone receptors, type of surgery, number of involved lymph nodes (LN) and stage of disease^22^. Some of these characteristics such as Her-2, estrogen receptors and progesterone receptors were eliminated from the study because of many cases (more than one third) of missing records.

### Preparing the data

"Foreign" package^23^ was used to convert the data, which stored in SPSS version 16 (Chicago, IL, USA), to R^24^, open source statistical software. When it was necessary, patients were stratified upon age group at diagnosis (<50 and ≥50 years), marriage status, tumor size (<2, 2-5 and >5 cm), number of involved LN (<2, 3-6, >7), stage of disease (I, II, III), type of surgery (breast conservative. lumpectomy, quadrantectomy, total Mastectomy) and metastasis status. It is worth noting that patients at diagnosis have no metastasis and it may develop during follow-up. Patients with missing records in characteristics selected for final model were eliminated from the study (81 (15.5%) patients).

### Statistical methods and variable selection

Kaplan-Meier estimator was used to predict the crude OS and estimates the median survival time in subgroups. For assessing the differences in survival distribution in subgroups, the log-rank test was used. CQR was used to provide in-depth insight in the multivariable association between prognosis factors and OS^25^. In this model, the *p*^th^ quantile (Q_p_) of true survival time (T) is:

Q_p_ = exp{X'β(*p*)}

where X is a vector of covariate and β(*p*) is a vector of coefficients for *p*^th^ quantile^21^. The estimation procedure in CQR is complex and complete details are described elsewhere^21^. Briefly, the coefficient of CQR was estimated by minimizing the below objective function.

**Figure:**
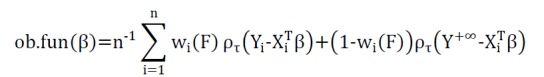


Where:

n: the number of observation

Y_i_: the observed survival time

Y^+∞^: an arbitrary and sufficiently large constant

X_i_: model matrix

β: coefficients

w_i_: the weight function

F: estimated locally Kaplan-Meier estimator

ρ_τ_(u): the quantile loss function for τ^th^ quantile = u.{τ-I(u<0)}

However, because there is no analytical method for estimating the variance of estimated coefficients, the resampling (bootstrap) method is used.

The CQR was fitted using the prognosis factors significant in log-rank test; in conclusion, age, tumor size and number of involved LN were selected for predicting (conditional) OS quantiles. In addition, because of probably non-linear effect of age on OS distribution of BC patients, the age was included in the model of the second order. Package "survival" was used to conduct the Kaplan-Meier and log-rank test^26^ and function LCRQ proposed by Wang and Wang^21^ was used to fit the CQR model.

## Results

**Figure 1:**
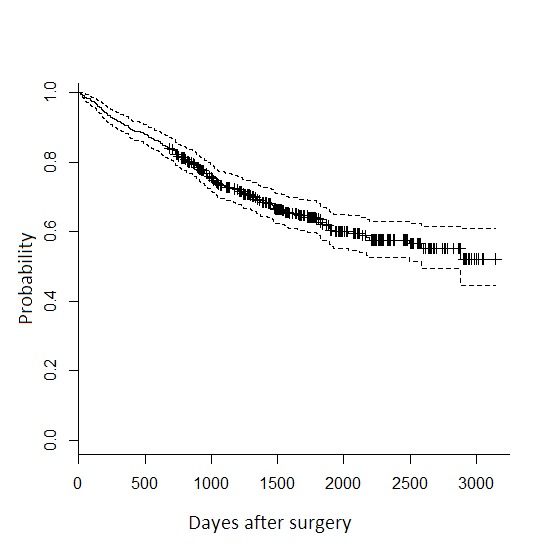
Plot of probability of surviving in BC patients after surgery.

Patients' follow-up ranged from about 3 to 197 months and median follow-up was 54.13 months. By the end of the study, 185 (35.4%) patients experience the death and 122 (23.4%) patients experience the metastasis or recurrence. One-, three-, and five-year survival rates were 90%, 73% and 62.5%, respectively that shows a constant decline over study period (Figure 1). Table 1 reports the patients and tumor characteristics and type of the received surgery. There was a crude relation between OS and age at diagnosis, tumor size, stage, metastasis status and number of involved LN (*P*<0.05).

As was mentioned in "statistical methods" subsection, the CQR model was used to explorer the multivariable relationship between prognosis factors and survival time (in month). Prognosis variables at diagnosis that showed a significant relationship with OS (Table 1), including standardized age (and age-squared), number of involved LN and tumor size were entered to the multivariable CQR model. Metastasis status and American Joint Committee on Cancer (AJCC) stage did not involve in CQR model, because the former showed the developed metastasis after the surgery and the latter had strong collinearity with number of involved LN and tumor size. The conditional median of survival time is:

$$Q_{0.5}=66.8-1.46\;age^{2}-4.97\;std.age-1.33Tsize-0.78nLN$$

**Table 1:** Patients, tumor and treatment characteristics of participates

			**Mean survival time (day)**		
**Characteristics**	**Patients, n (%)**	**Deaths, n (%)**	**Mean**	**SE**	***P*** ** value**
Age at diagnosis (yr)					0.001
<50	351 (67.2)	109 (31.1)	3330	317.0	
≥50	171 (32.8)	76 (44.4)	2370	160.9	
Marital status					0.867
Single	21 (4.0)	6 (28.6)	2494	255.1	
Married, divorced, widowed	501 (96.0)	179 (35.7)	3128	267.9	
Tumor size					0.001
<2 cm	324 (62.1)	91 (28.1)	4161	184.3	
2-5 cm	130 (24.9)	46 (35.4)	2898	201.9	
≥5 cm	68 (13.0)	48 (70.6)	1889	154.1	
Involved lymph node					0.001
≤2	212 (52.2)	54 (25.5)	2817	162.7	
3-6	110 (27.1)	33 (30.0)	2485	110.8	
≥7	84 (20.7)	43 (51.2)	2103	139.7	
Metastasis status					0.001
Present	122 (23.4)	47 (38.5)	2581	056.5	
Absence	400 (76.6)	290 (72.5)	2368	216.5	
AJCC stage ^a^					0.001
I	194 (39.5)	43 (22.2)	2602	076.9	
II	69 (14.1)	23 (33.3)	2520	121.9	
III	107 (21.8)	36 (33.6)	2436	114.8	
Unknown	122 (24.6)	74 (61.2)	2388	218.9	
Type of surgery					0.843
Breast conserving	26 (5.0)	8 (30.8)	3294	353.9	
Lumpectomy	392 (75.1)	145 (37.0)	3011	304.4	
Quadrantectomy	41 (7.9)	15 (36.6)	2437	198.7	
Total mastectomy	63 (12.0)	17 (27.0)	2336	147.2	

^a^ American Joint Committee on Cancer stage

Which in the above mentioned expression, std. age, age2, Tsize and nLN stand for standardized age, age-squared, tumor size and number of involved LN, respectively. Table 2 shows the estimated coefficients of 1, 2.5, 5, 10, 25, 50, 75, 90, 95, 97.5 and 99^th^ conditional quantile of survival times. Using the estimated CQR model presented in Table 2, quartile of survival time estimated for 18 different scenarios were calculated. These sceneries were defined using three different ages (40, 45 and 50 yr), two different number of involved LN (3 and 6) and three different tumor sizes (1.5, 3.5 and 6 cm). Table 3 shows the estimated conditional quartiles of time to death (in month) using the CQR for these eighteen scenarios. The higher age at diagnosis, the more number of involved LN and/or larger tumor size, led to decreasing the median and other quartile of OS distributions (Table 3).

**Table 2:** Estimated coefficient and its standard error (in parenthesis) of multivariable censored quantile regression of survival times

**Quantiles**	**Constant**	**Age-squared**	**Standard age** ^†^	**Tumor size**	**Involved lymph node**
1	2.7	-1.2 (0.75)	1.5 (1.67)	0.5 (1.03)	-0.03 (0.3)
2.5	9.2	-1.3 (0.94)	0.2 (1.89)	-0.8 (1.02)	-0.1 (0.27)
5	14.1	-1.2 (1.06)	-1.1 (2.33)	-1.3 (0.42)	-0.3 (0.15)
10	25.1	-1.6 (0.75)	-4.0 (2.34)	-2.5 (1.09)	-0.4 (0.59)
25	45.7	-0.5 (2.62)	-5.2 (2.69)	-3.2 (1.40)	-0.4 (0.53)
50	66.8	-1.5 (0.72)	-5.0 (3.30)	-1.3 (0.72)	-0.8 (0.48)
75	88.7	-3.9 (1.76)	-2.2 (3.81)	-1.2 (3.83)	-0.6 (0.27)
90	92.7	-0.8 (2.65)	-3.1 (1.48)	0.4 (2.28)	0.2 (0.61)
95	99.4	-1.0 (1.72)	-2.3 (3.40)	-0.4 (0.28)	0.2 (0.47)
97.5	102.6	-1.4 (1.52)	-1.8 (2.41)	-0.9 (1.84)	0.1 (0.50)
99	107.2	-1.8 (1.58)	-1.3 (3.02)	-1.42 (1.5)	-0.2 (0.52)

Bold figures: significant at 0.05

**Table 3:** Estimated quantiles of survival time distribution for different sceneries of age, number of involved lymph node and tumor size combinations

**Tumor size**	**1.5 cm**						**3.5 cm**						**6 cm**					
**No. ILN** ^a^	**3**			**6**			**3**			**6**			**3**			**6**		
Age (yr)	40	45	55	40	45	55	40	45	55	40	45	55	40	45	55	40	45	55
Q 25	42.91	40.25	36.22	41.80	39.14	35.11	36.49	33.82	29.78	35.36	32.70	28.72	28.42	25.76	21.73	27.31	24.65	20.62
Q 50	65.09	62.90	58.71	62.73	60.59	56.36	62.40	60.28	56.04	60.09	57.94	53.70	59.01	56.95	52.71	56.76	54.61	50.73
Q 75	85.16	85.55	82.35	83.51	83.92	80.70	82.84	83.23	80.04	81.19	81.58	78.39	79.94	83.23	80.04	78.29	81.58	78.39

^a^ Number of involved lymph node

## Discussion

The main aim of this study was to investigate factors affecting the distribution of OS in patients with non-metastatic BC. For the above-mentioned aim, a distribution-free CQR model introduced by Wang and Wang^21^ was used. The CQR model has many interesting features. This model is distribution-free but give complete information about distribution of time-to-event^18,21^. In addition, the assumption of random censoring that is important in Cox proportional hazard model was relaxed in this model^17,21^. Another feature of CQR is the direct interpretation of estimated effects in terms of change in quantile of survival time distribution^17,21^. Our results revealed that, comparing to the studies in developed western countries^3^, patients participants in this study, were diagnosed at the later age (about one-third were diagnosed at age ≥ 50 yr), and later stages (about 40% were diagnosed with tumor size ≥ 2 cm, about 37% with number of involved LN ≥ 2 and about 53% at stage II/III). This maybe because of lack of awareness about the signs of BC, lack of participants in screening program and lack of (inadequateness) performing of regular breast self-examination ^3,27^ . On the other hand, the late diagnosis of BC at advanced level could be cause to reduction in OS (Table 2). Unfortunately, in Iran, women have no sufficient knowledge about the breast cancer symptoms, breast self-examination, clinical examination and mammography and about 83% of women do not perform regular monthly breast self-examination^27^. In addition to the late diagnosis, it seems that the socio-economics and race are important factors affecting –survival- of BC patients^28,29^.

Age, tumor size, number of involved LN, stage of disease, progesterone receptor status and human epidermal receptor are important prognosis factors at diagnosis and, in addition, loco-regional metastasis is an important intermediate prognosis factor of unadjusted OS (Table 1). This result is consistent with another study as for the role of these risk factors^13^.

To the best of our knowledge, there are only limited studies addressing the distribution of OS in BC patients and its related factors^21^. The multivariable analysis of CQR showed that age at diagnosis, tumor size and number of involved LN are important factors in determination the distribution of patient's lifetime at diagnosis. Based on developed model, the median, first and 3^rd^ quartiles of OS decrease by increasing of age in all subgroups. Such conclusion is showed by number of involved LN and tumor size. This finding supports the important role of early detection of BC and is in concordance with other studies^13^.

On the other hand, an important finding that CQR model revealed is that the change in distribution of (log) survival time may not preserve the distribution family. Change in the distribution of other characteristics by the change of covariate has been reported earlier^30^. Therefore, in this situation, application of parametric survival models may lead to invalid and biased conclusion.

In this study, there were some limitations on the data and the statistical method. Unfortunately, there are many non-responses in patients' medical records, especially in some important features including estrogen and progesterone receptors, as well as, Her-2 and p53. On the other hand, in the CQR that used in this study, all independent variables should be of quantitative type. Therefore, if there are one or more qualitative variables, a stratified analysis should be used.

## Conclusions

This study confirms the importance of early detection of BC and warns on the possible changes in distribution family of survival time.

## Acknowledgments

This article extracted from MSc dissertation supported by Deputy of Research and Technology of Hamadan University of Medical Sciences. Authors would like to thanks this deputy and would like to thanks Hamadan Mahdieh Oncology Center’s personnel for their corporation.

## Conflict of interest statement

None declared.
